# M^6^A-mediated upregulation of HOXC10 promotes human hepatocellular carcinoma development through PTEN/AKT/mTOR signaling pathway

**DOI:** 10.1007/s12672-023-00786-0

**Published:** 2023-09-21

**Authors:** Miao Li, Qianwen Guo, Qian Shi, Yanzhi Rao, Yixin Dong, Fangjie Chen, Xun Qi

**Affiliations:** 1 Department of Microbiology and Parasitology, College of Basic Medical Sciences, China Medical University, Shenyang, 110122 China; 2 Key Laboratory of Diagnostic Imaging and Interventional Radiology of Liaoning Province, Department of Radiology, The First Hospital of China Medical University, 155 Nanjing Bei Street, Shenyang, 110001 China; 3 Department of Medical Genetics, School of Life Sciences, China Medical University, No.77 Puhe Road, Shenyang, 110122 China

**Keywords:** HOXC10 HCC proliferation M6A PTEN, AKT, mTOR pathway

## Abstract

Human Hox genes (Homeobox) play a crucial role in embryonic development and cancer. The HOXC10 gene, a member of the HOX family, has been reported abnormally expressed in several cancers. However, the association between HOXC10 and hepatocellular carcinoma (HCC) remains to be elucidated. In the present study, tissue microarray cohort data showed that high levels of HOXC10 expression predicted a poor survival in HCC patients. Meanwhile, HOXC10 was significantly upregulated in the Huh7 cell line compared with the well differentiated cell line HepG2 and human normal liver cells. Functionally, silencing HOXC10 in Huh7 cells inhibited cell proliferation, increased apoptosis, and inhibited invasion and migration of HCC cells. HOXC10 overexpression in HepG2 cells increased cell proliferation, decreased apoptosis, and increased invasion and migration of HCC cells. In the HepG2 xenograft models, HOXC10 increased the tumor volume and weight compared with control. Mechanistically, the m6A modification of HOXC10 by METTL3 enhanced its expression by enhancing its mRNA stability. Both the in vitro and in vivo results showed that overexpressed HOXC10 activated the PTEN/AKT/mTOR pathway. In summary, the findings highlight the importance of HOXC10 in the regulation of HCC progression. HOXC10 is potentially a future therapeutic target for HCC treatment.

**Supplementary Information:**

The online version contains supplementary material available at 10.1007/s12672-023-00786-0.

## Highlights

- HOXC10 is a potential prognostic biomarker for HCC patients.
- HOXC10 upregulation in HCC could promote cell proliferation, migration and invasion.
- HOXC10 function in HCC cells might be associated with the modulation of PTEN/AKT/mTOR signaling pathway.
- M6A modification of HOXC10 by METTL3 enhanced its expression by enhancing its mRNA stability.

**Supplementary Information:**

The online version contains supplementary material available at 10.1007/s12672-023-00786-0.

## Background

Primary liver cancer is the seventh most frequently occurring cancer worldwide and the second most common cause of cancer death [1]. Globally, hepatocellular carcinoma (HCC) is the leading type of liver cancer, accounting for approximately 75% of all liver cancers [2]. Despite recent advances in cancer therapeutic strategies, the overall survival rate of patients with HCC remains unsatisfactory, mainly due to its insidious onset and high rate of recurrence [3]. Therefore, an urgent need remains for novel and effective therapies.

Homeobox genes (HOX) encode transcription factors involved in cell differentiation and embryonic development [4]. Mammalians have 39 HOX genes organized in four clusters (A–D), locating on chromosomes 7, 17, 12 and 2, respectively [5, 6]. The HOXC10 gene belongs to the HOXC cluster and is located on chromosome 12, which contains an intron and two exons in its gene sequence [7]. In addition, it has been recently reported that HOXC10 plays an important role in the development of multiple cancers, including breast cancer, osteosarcoma, glioma and thyroid cancer [8–11]. Our previous study found that HOXC10 was a novel oncogene in NSCLC cells [12]. However, HOXC10 expression and function in HCC are relatively unknown.

In the current study, we first determined the effect and significance of HOXC10 expression on HCC prognosis. We also used two HCC cell lines and mice models to analyze the biologic functions and potential molecular mechanisms in cancer progression. Importantly, we proved that the m6A modification of HOXC10 by METTL3 enhanced its expression by enhancing its mRNA stability and HOXC10 might activate the PTEN/AKT/mTOR signaling pathway. Thus, HOXC10 is potentially a therapeutic target for HCC.

## Materials and methods

### Tissue microarray and immunohistochemistry

The HCC tissue microarray (HLivH180Su18) was obtained from Outdo Biotech Co., Ltd. (Shanghai, China). The HLivH180Su18 TMA contained 76 HCC tissues, which was used as the exploring cohort for the detection of HOXC10 expression and relationships between HOXC10 expression and clinicopathological parameters. The study was approved by the Ethics Committe of Shanghai Outdo Biotech Company. Immunohistochemistry (IHC) studies of HOXC10 were performed on HCC samples of tissue microarray. IHC was performed as previously described [13]. Rabbit anti-human polyclonal HOXC10 antibody (Abcam, Cambridge, MA, USA) was used at a 1:500 dilution. The proportion of positively stained tumor cells was graded as: 0 (no positive tumor cells), 1 (< 10%), 2 (10–50%), or 3 (> 50%). The intensity of staining was scored as 0 (no staining), 1 (weak), 2 (moderate), or 3 (strong). Total scores equal the percentages of positive cells multiplied by staining intensity. Total scores ≤ 45% were deemed to be the low expression and total scores > 45% were deemed to be the high expression.

The IHC staining of Ki-67 was performed in xenograft tissue. Tissue samples were embedded in paraffin and cut into 5 µm sections, which were deparaffinized in xylene, rehydrated through graded ethanol, quenched for endogenous peroxidase activity in 3% hydrogen peroxide, and processed for antigen retrieval by microwave heating for 7 min in 10 mM citrate buffer (pH 6.0). The sections were then sequentially incubated with primary antibodies against KI67 Polyclonal antibody (1:2000, PROTEINTECH, Wuhan, Hubei, P.R.C.) overnight at 4 °C in a humified chamber, and appropriate secondary antibodies for 1 h. Finally, the sections were stained with 3,3-diaminobenzidine tetrahydrochloride for 5 min at RT, and imaged under a microscope.

### Cell culture

The two human HCC cell lines Huh7 and HepG2, a human normal liver cell line LO2, were purchased from the American Type Culture Collection (Manassas, VA, USA). Cells were maintained in DMEM (Invitrogen, Carlsbad, CA, USA) supplemented with 10% fetal bovine serum (FBS, GIBCO Carlsbad, CA, USA), 100 U/mL penicillin, and 100 mg/mL streptomycin in a humidified environment at 37 °C with 5% CO2.

### RNA isolation and RT-qPCR

Total RNA was isolated using TRIzol (Invitrogen) and then converted to cDNA with the PrimeScript RT reagent kit (Takara, Dalian, China) according to the manufacturer's instructions. Real-time PCR was performed using the SYBR Primix kit (Takara). The expression level was calculated using the 2-ΔΔCt method and normalized to GAPDH expression. Primers are described in Table S1.

### Gene set enrichment analysis (GSEA)

The transcriptome data of 373 patients with HCC was downloaded from The Cancer Genome Atlas (TCGA) https://portal.gdc.Cancer.gov/. TCGA is an open source database and does not require additional ethical approval. We complied with the rules for the acquisition and utilization of data. The 373 patient samples were divided into two expression level groups based on the median expression values of HOXC10 and PTEN, respectively. Then the differential gene expression analysis of the two groups was carried out, and the two groups of HOXC10 differential gene list and PTEN differential gene list were obtained. GSEA were run and the cut-off criteria were as follows: normalized enrichment scores (NES) > 1.0 and nominal p < 0.05. We observed the enrichment of Hoxc10 and PTEN in mTOR-related pathway.

### Western blotting

The proteins were separated using electrophoresis on 10% SDS-PAGE and then electrophoretically transferred onto polyvinylidene difluoride (PVDF) membranes (Invitrogen). The membranes were then incubated with the following primary antibodies: anti-HOXC10 (Abcam, Cambridge, MA, USA), anti-PTEN, anti-AKT, anti-p-AKT, anti-mTOR (Cell Signaling Technology, Beverly, MA, USA), and anti-GAPDH (Proteintech, Wuhan, China). Next, the membranes were incubated with horseradish peroxidase-conjugated goat anti-rabbit antibody (Abcam, Cambridge, MA, USA) and the protein bands were examined using ECL Western blotting Substrate (Thermo Fisher Scientific, Waltham, MA, USA).

### Generation of HOXC10 stable expression cell lines and RNA interference

The constructed HOXC10 overexpression plasmids were prepared as previously described [12]. Western blotting was used to verify the establishment of HepG2 cells with stable overexpression of HOXC10. The human HOXC10-specific siRNAs were purchased from GenePharma (Shanghai, China). The siRNA sequences are listed in Table S1. All siRNAs were transfected into Huh7 cells using Lipofectamine™ RNAiMAX (Thermo Fisher Scientific) according to the manufacturer's instructions.

### Proliferation analysis

Cell proliferation was assayed using the Cell Counting Kit-8 (CCK-8). Huh7 and HepG2 cells were plated into 96-well plates at a density of 4 × 10^3^/well. CCK-8 solution was added to each well at 0, 24, 48, and 72 h after siRNA transfection. After incubation for 2 h at 37 °C, the absorbance value at 450 nm was determined using a microplate reader.

For colony formation assay, cells (3.5 × 10^5^ cells per well) were seeded in a 6-well plate and incubated for 2 weeks at 37 °C. Then, cells were washed twice in PBS, fixed with 4% formaldehyde for 15 min and stained for 10–30 min with crystal violet. The colonies were counted in triplicate assays.

### Cell apoptosis

Cell apoptosis was performed using Annexin V-allophycocyanin/Propidium Iodide (PI) Apoptosis Detection kit (KeyGEN BioTECH, Nanjing, China). The stained cells were examined using flow cytometry (BD, Biosciences, San Jose, CA, USA).

### Migration and invasion assays

Cell migration and invasion were detected using Transwell chambers with 8-μm polycarbonate nucleopore filters (Millipore, Bedford, MA, USA). In brief, 5 × 10^4^ transfected cells in 200 µL serum-free medium were added to the upper compartment of 24-well Transwell culture chamber. The lower compartment was loaded with 600 µL complete medium. After 24 h of incubation, cells that had invaded the bottom surface of the transwell were fixed with 4% paraformaldehyde (PFA), stained with crystal violet, and counted microscopically (Olympus, Tokyo, Japan). For the invasion assay, the upper compartment was precoated with 100 μl of Matrigel (BD Biosciences). All other processes were the same as for the transwell migration assay according to the method described by the manufacturer.

### In vivo tumorigenesis in nude mice

All animal studies were conducted in accordance with the principles and procedures outlined in the Wuhan Servivebio of China Guide for the Care and Use of Animals. The study was approved by the Ethics Committe of Wuhan Servivebio. First, 6.25 × 10^6^ HepG2 cells transfected with stable HOXC10-overexpressing cells and the control vector in 0.1 mL DMEM were subcutaneously injected into the right symmetric flank of 4–6-week-old male BALB/c mice. Tumor size was measured with calipers every 3 days and the tumor volume calculated using the following formula: 0.5 × length × width^2^. The mice were sacrificed after 21 days and the tumor tissues excised and weighted.

### M^6^A-qPCR

The MeRIP assay was conducted using a Magna RIP RNA-binding protein immuno-precipitation kit (Millipore, Billerica, MA, USA) according to protocols. Primers used for m6A-qPCR were listed in Table S1.

### RNA stability assays

Cells were seeded in 12-well plates overnight, and then treated with actinomycin D (10 μg/mL, HY-17559, MedChem Express). After incubation for 2, 4 and 6 h, the cells were harvested and RNA was extracted to detect the stability of HOXC10 using RT-qPCR.

### Statistical analysis

All statistical analyses were performed using SPSS 20.0 Statistics (IBM, Armonk, NY, USA). Statistical significance between groups was assessed using Student’s *t*-test or Mann–Whitney *U* test. Multiple comparisons were performed using ANOVA. P-values < 0.05 were considered statistically significant.

## Results

### HOXC10 expression in HCC tissues and positively correlates with poor prognosis

**Fig. 1:**
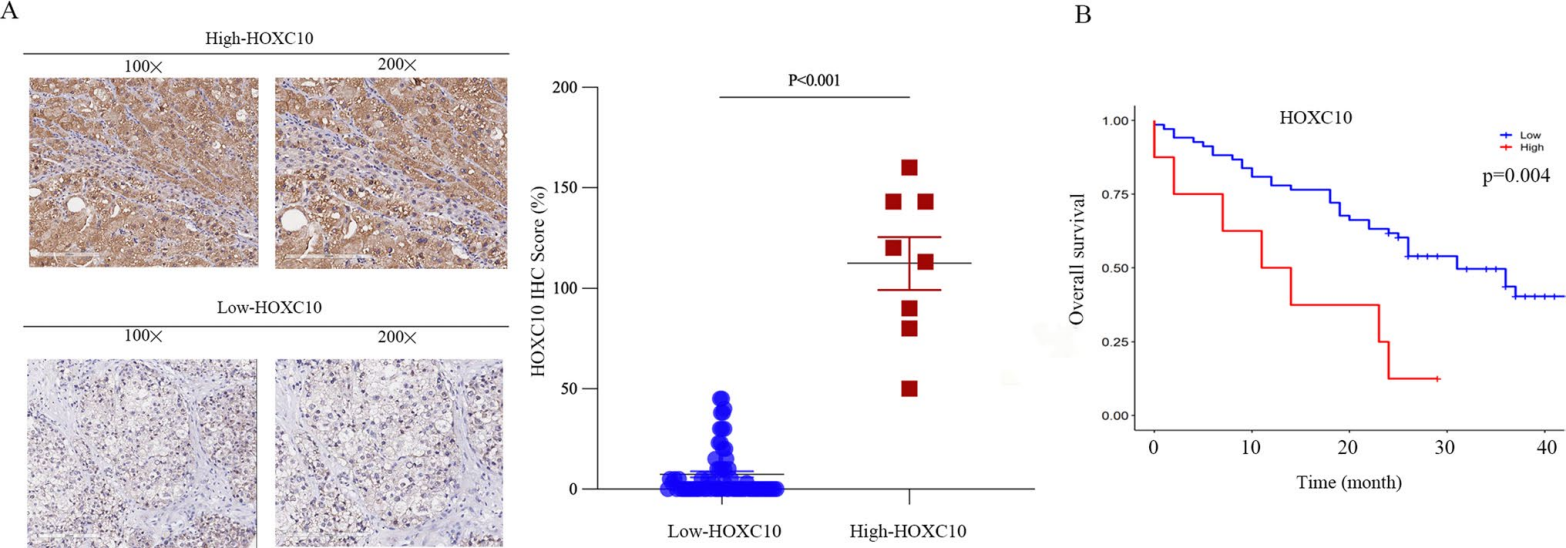
HOXC10 expression in HCC tissues. **A** HOXC10 expression in HCC by IHC. **B** Relationships between HOXC10 expression in HCC and overall survival (OS). Kaplan–Meier analysis of OS (P = 0.004) according to HOXC10 levels

To confirm the clinical relevance of HOXC10 induction in HCC, we performed IHC staining with a validated antibody against HOXC10 on 76 HCC samples (Fig. 1A). HOXC10 was expressed at higher levels in 8 cases (10.53%), while it was lower in 68 cases (89.47%). The correlation of HOXC10 expression with clinicopathological characteristics in tissue microarray of 76 HCC patients is further summarized in Table 1. Kaplan–Meier method for overall survival (OS) indicated that the patients with low HOXC10 expression had significantly better survival rates compared to patients with high HOXC10 expression (Fig. 1B). Univariate analysis revealed that HOXC10 expression (P = 0.007), Tumor size (P = 0.024), Grade stage (P = 0.032), TNM stage (P = 0.017) and T stage (P = 0.017) were significant prognostic factors for OS. Multivariate analysis further indicated HOXC10 expression as an independent prognostic factor (HR:7.145; 95% CI: 1.665–30.660; P = 0.008) (Table 2).

**Table 1:** Correlation between HOXC10 expression and clinicopathological characteristics

	variables	HOXC10 expression		total	χ^2^	p value
		low	high			
Age (year)					4.459	0.035
	≤ 54	40	1	41		
	> 54	28	7	35		
Sex					0.000	1.000
	male	57	7	64		
	Female	11	1	12		
Grade					0.001	0.970
	I/II	23	1	24		
	III	21	2	23		
T stage					0.205	0.651
	T1/T2	32	5	37		
	T3/T4	36	3	39		
TNM stage					1.185	0.276
	I/II	35	6	41		
	III	27	1	28		
Tumor size					0.205	0.651
	≤ 6 cm	32	5	37		
	> 6 cm	36	3	39

**Table 2:** Univariate and multivariate analyses of the factors correlated with Overall Survival

Variables	Univariate analysis		Multivariate analysis	
	HR (95%CI)	p value	HR (95%CI)	p value
HOXC10	3.136(1.369–7.185)	0.007	7.145(1.665–30.660)	0.008
Age	0.732(0.397–1.350)	0.318	–	n.a
Sex	0.403(0.143–1.134)	0.085	–	n.a
Tumor size	2.047(1.101–3.807)	0.024	1.595(0.499–5.101)	0.431
Grade stage	2.281(1.074–4.847)	0.032	1.473(0.602–3.604)	0.396
TNM stage	2.225(1.152–4.295)	0.017	1.858(0.576–6.001)	0.300
T stage	2.225(1.152–4.295)	0.017	1.858(0.576–6.001)	0.300

n.a. reprensents not applicable

### Elevated expression of HOXC10 in HCC cells

**Fig. 2:**
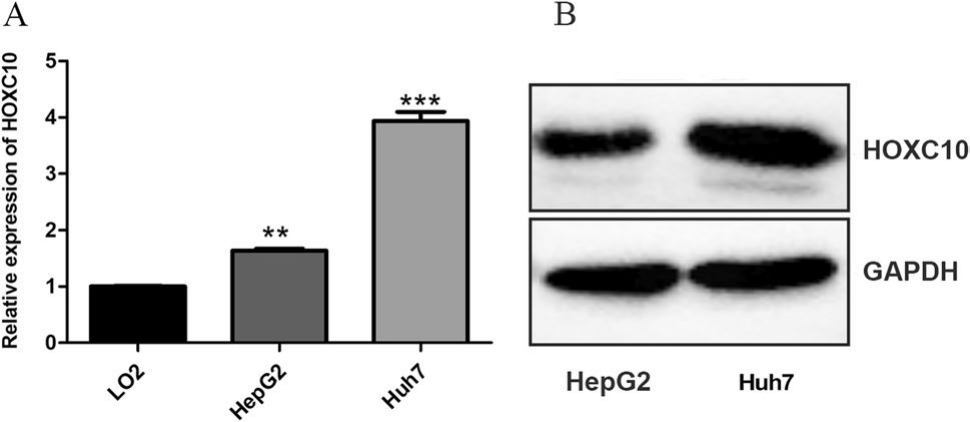
Expression of HOXC10 in HCC cells.** A** HOXC10 mRNA expression in HCC cell lines based on RT-qPCR. **B** Immunoblotting was used to assess and compare HOXC10 protein levels between HepG2 and Huh7 cells. Data shown are mean ± SD, **P < 0.01

RT-qPCR was performed to quantify the HOXC10 expression levels in HCC cell lines and a human normal hepatic cell line LO2. As shown in Fig. 2A, HOXC10 was more highly expressed in Huh7 cells and less expressed in the well differentiated cell line HepG2 and the human normal liver cell line LO2. Western blotting confirmed that HOXC10 was well expressed in Huh7 cells and moderately expressed in HepG2 cells (Fig. 2B).

### HOXC10 promotes HCC cell proliferation in vitro

**Fig. 3:**
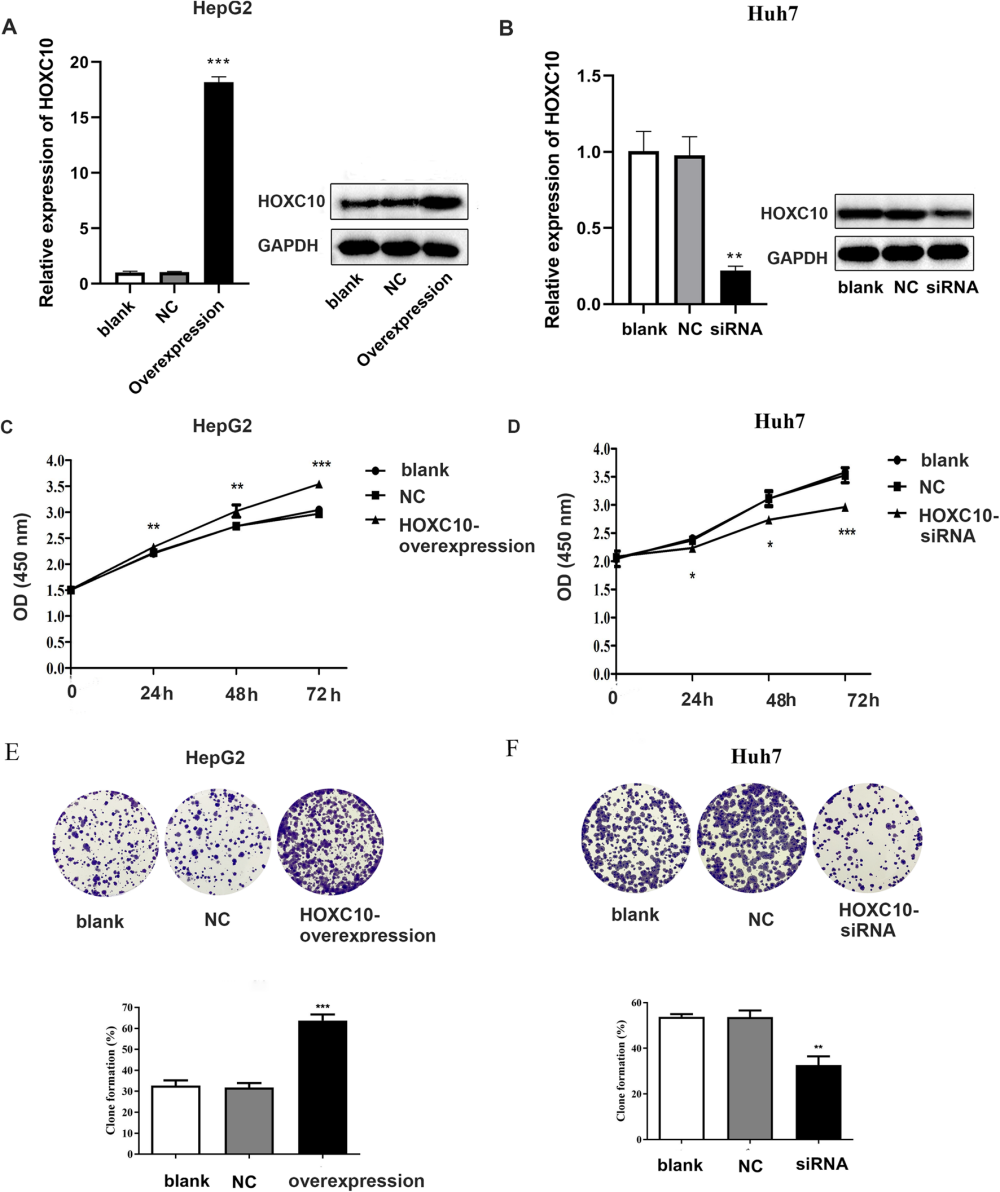
HOXC10 promotes HCC cell proliferation. **A** RT-qPCR and western blotting analysis of HOXC10 after HOXC10 overexpression in HepG2 cells. **B** RT-qPCR and western blotting analysis of HOXC10 after HOXC10 knockdown in Huh7 cells. **C** Cell viability of HepG2 after HOXC10 overexpression was examined at 0, 24, 48, and 72 h using CCK8 assays. **D** Cell viability of Huh7 cells after HOXC10 knockdown was examined at 0, 24, 48, and 72 h using CCK8 assays.** E**–**F** Colony formation assays were used to assess the role of HOXC10 overexpression in HepG2 cells and HOXC10 siRNA in Huh7 cells. Data shown are mean ± SD, *P < 0.05, **P < 0.01, ***P < 0.001 (compared with negative control)

To explore the role of HOXC10 on cell viability, the HOXC10 expression was stably upregulated in the HepG2 cells using a lentivirus system and the HOXC10 siRNA was transfected into Huh7 cells. The efficiency of transfection was verified based on RT-qPCR and western blotting analyses (Fig. 3A, B). CCK8 and colony formation assays were used to assess the role of HOXC10 in HCC proliferation. Compared to the negative control, HOXC10-overexpressing HepG2 cells showed a increase in cell viability. In addition, knockdown of HOXC10 significantly reduced viability in Huh7 cells compared with the control group (Fig. 3C–F).

### HOXC10 inhibits apoptotic cell death in HCC cells in vitro

**Fig. 4:**
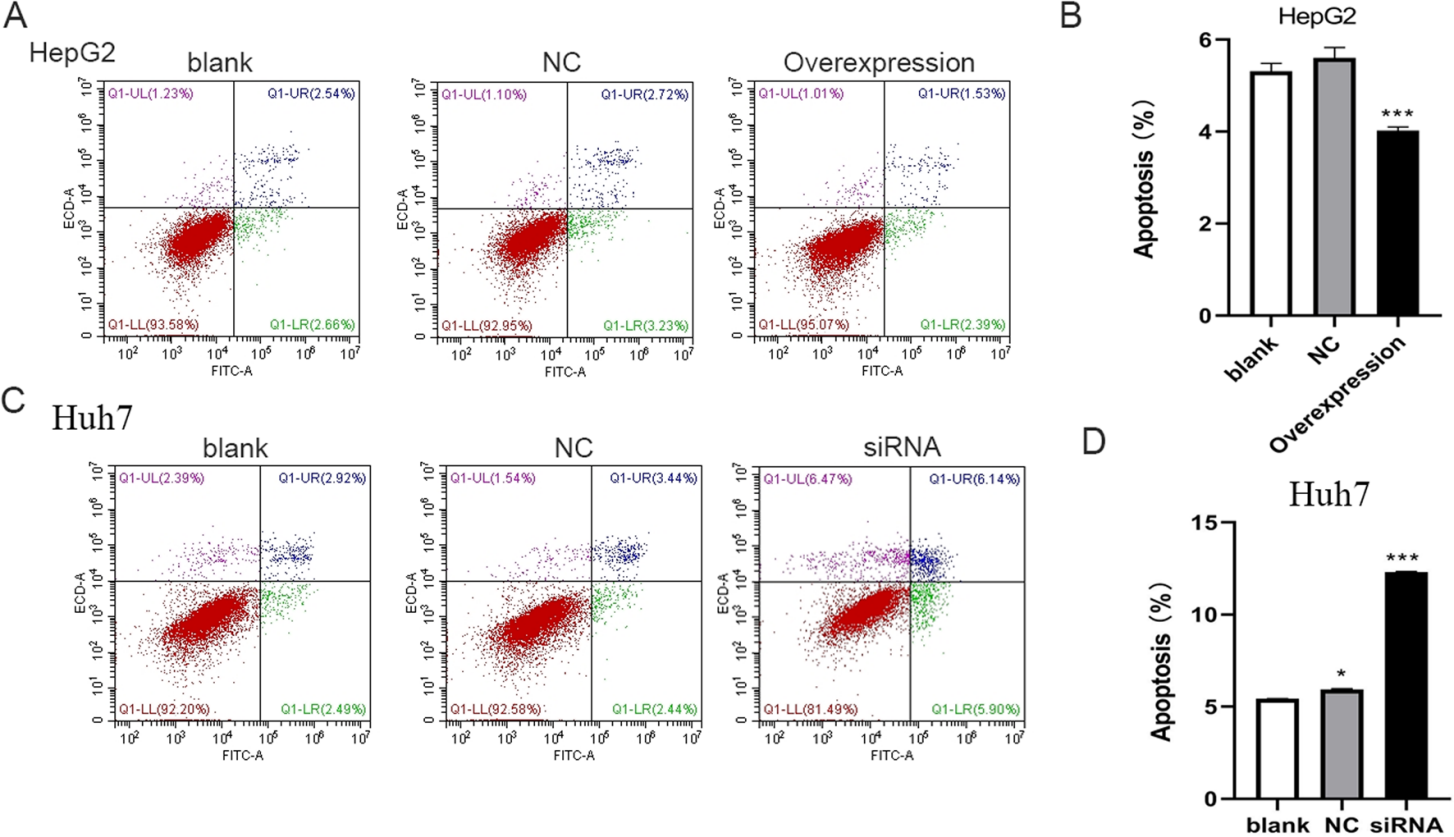
HOXC10 inhibits apoptotic cell death in HCC cells. Cell apoptosis was examined at 48 h after HOXC10 overexpression or knockdown using Annexin V/PI staining and flow cytometry. **A-B** HOXC10 overexpression in HepG2 significantly decreased the percentage of apoptotic cells. **C-D** HOXC10 knockdown in Huh7 cells increased the percentage of apoptotic cells. Data shown are mean ± SD, *P < 0.05, **P < 0.01, ***P < 0.001 (compared with negative control)

We hypothesized that increased cell viability inhibits cancer cell apoptosis. Using Annexin V-PI Apoptosis Detection Kit, overexpression of HOXC10 caused a 28.5% decrease in apoptosis (Fig. 4A, B). However, knockdown of HOXC10 in Huh7 cells induced a 53.3% increase in apoptosis compared with the control group (Fig. 4C, D).

### HOXC10 promotes cell migration and invasion in vitro

**Fig. 5:**
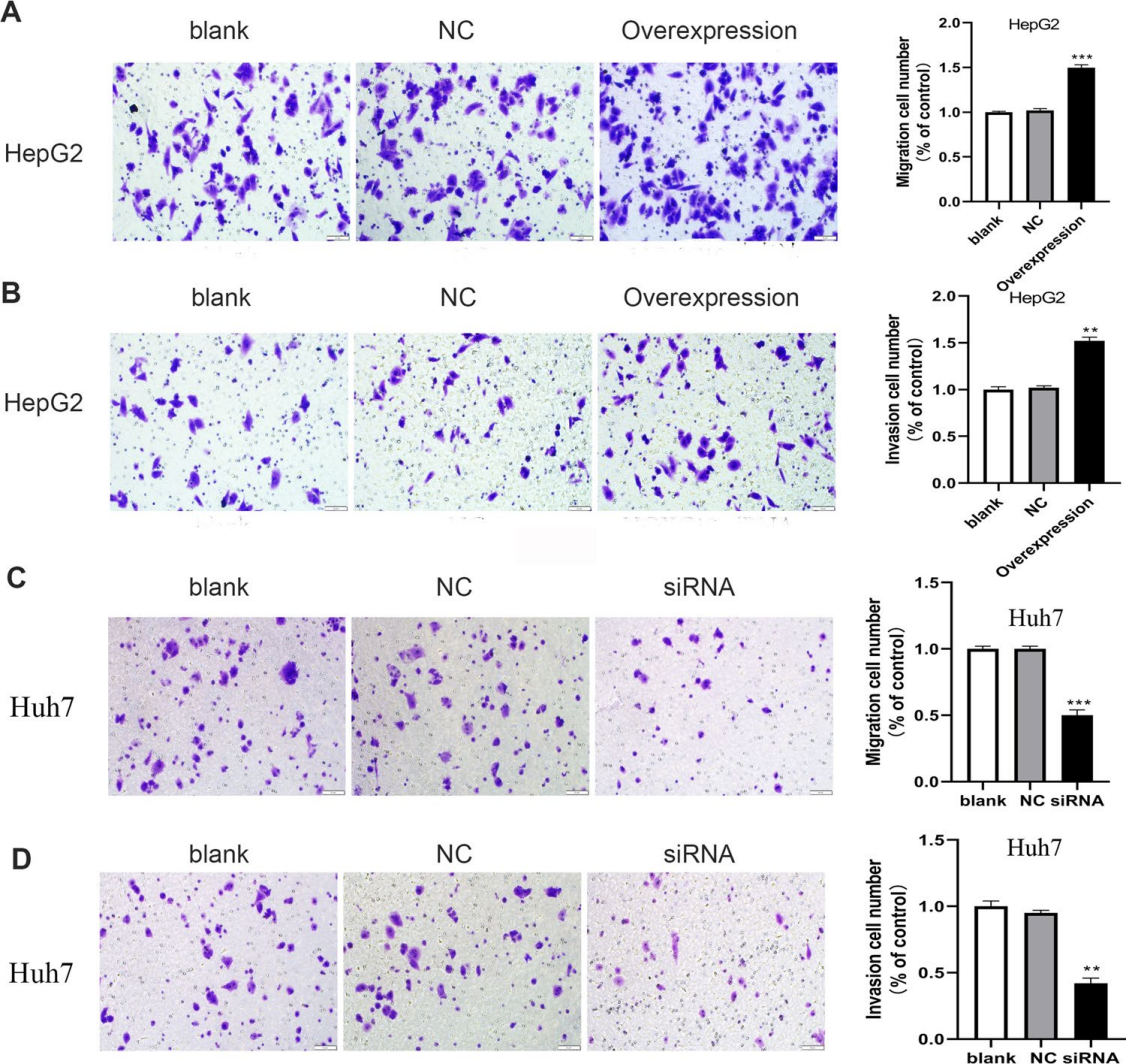
HOXC10 promotes cell migration and invasion. **A-B** Cell migration and invasion of HepG2 cells after HOXC10 overexpression were measured using the Transwell and Matrigel assays. **C-D** Cell migration and invasion of Huh7 cells after HOXC10 knockdown were measured using the Transwell and Matrigel assays. Data shown are mean ± SD, *P < 0.05, **P < 0.01, ***P < 0.001 (compared with negative control)

The transwell migration and invasion assays showed that HOXC10 overexpression significantly enhanced the migration and invasion abilities of HepG2 cells (Fig. 5A, B). Consistent with previous findings, downregulating the expression of HOXC10 using siRNA suppressed the migration and invasion abilities of Huh7 cells in vitro (Fig. 5C, D).

### METTL3 induced HOXC10 m^6^A to enhance its mRNA stability

**Fig. 6:**
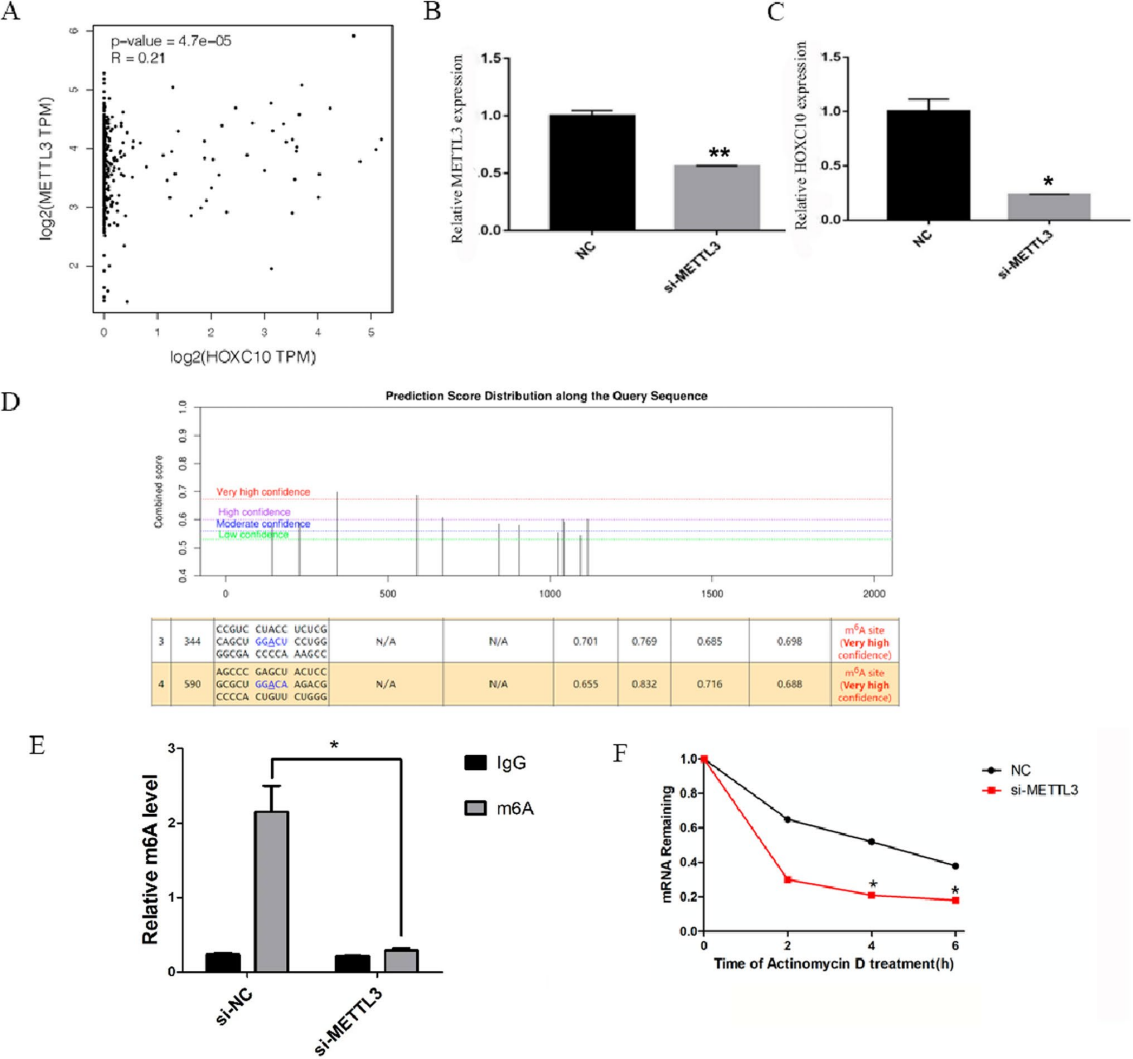
METTL3 induced HOXC10 m^6^A to enhance its mRNA stability in Huh7 cells. **A** The correlation of METTL3 and HOXC10 in HCC based on GEPIA database. **B-C** Expression of METTL3 and HOXC10 was tested by RT-qPCR when transfected si-METTL3 in Huh7 cells. **D** Identify the specific m^6^A methylation loci of HOXC10 by SRAMP website. **E** M^6^A level in HOXC10 in Huh7 cells with si-NC or si-METTL3 by MeRIP-qRCR. (F) HOXC10 mRNA stability detected by RT-qPCR.*P < 0.05; **P < 0.01; ***P < 0.001

Based on GEPIA database analysis, the HOXC10 expression was positive with METTL3 in HCC (Fig. 6A), indicating that HOXC10 might be regulated by METTL3 in an m^6^A pattern. RT-qPCR indicated that knockdown of METTL3 leads to a decrease HOXC10 in Huh7 cells (Fig. 6B, C). To identify the specific m^6^A methylation loci of HOXC10, we applied the SRAMP website indicating that some positions existed abundance of m6A methylation loci (Fig. 6D). M^6^A RIP-qPCR analysis indicated that m^6^A was highly enriched within HOXC10 in Huh7 cells and METTL3-induced HOXC10 m^6^A hyper-methylation in Huh7 cells (Fig. 6E). Moreover, ActD assay showed that METTL3 loss labilized HOXC10 mRNA in Huh7 cells (Fig. 6F). Above results indicated that METTL3-induced HOXC10 m^6^A hypermethylation to enhance its mRNA stability in HCC.

### HOXC10 activates the PTEN/AKT/mTOR signaling pathway

**Fig. 7:**
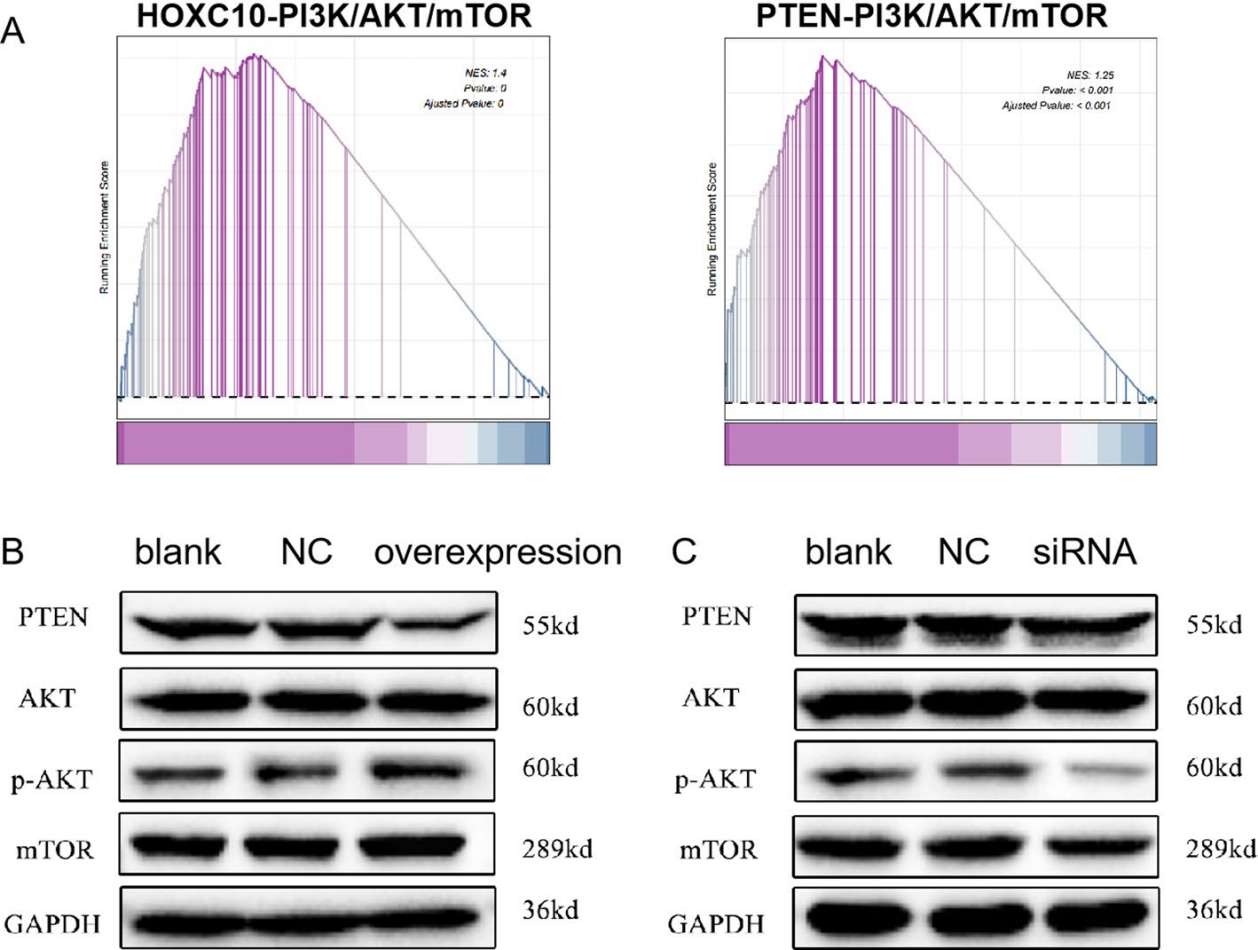
HOXC10 activates PTEN/AKT/mTOR signaling pathway. **A** The enrichment of HOXC10 and PTEN in mTOR-related pathway were Analyzed by GSEA. **B** PTEN, AKT, p-AKT, and mTOR expression were detected using western blotting in HOXC10-overexpressing HepG2 cells. **C** PTEN, AKT, p-AKT, and mTOR expression were detected using western blotting in HOXC10-knockdown Huh7 cells

To determine the potential mechanism for HOXC10 regulation of HCC, the main signaling pathways disturbed by HOXC10 alteration in HCC were searched. PTEN is an upstream regulator of the protein kinase B/mammalian target of rapamycin (AKT/mTOR) signaling pathway [14], and the AKT/mTOR pathway correlates with tumor proliferation, survival, apoptosis, and invasion/metastasis [15, 16]. Then the enrichment of HOXC10 and PTEN in mTOR-related pathway were Analyzed by GSEA. The results were shown in Fig. 7A, the NES values for HOXC10 and PTEN in the PI3K/AKT/mTOR pathway were 1.4 and 1.25 (*p* < 0.001). The results suggested that HOXC10 and PTEN are significantly high correlated with the activation of PI3K/AKT/mTOR signaling pathway. Thus, the effects of HOXC10 on the PTEN/AKT/mTOR pathway were investigated by western blotting analysis. As shown in Fig. 7B, western blotting analysis confirmed decreased PTEN and increased phosphorylation of AKT and mTOR in HOXC10 overexpression compared with vector control HepG2 cells. Furthermore, the AKT/mTOR pathway was inhibited in Huh7 cells transfected with HOXC10 siRNA (Fig. 7C). These results indicated that HOXC10 might activate the PTEN/AKT/mTOR signaling pathway.

### HOXC10 accelerates hepatic tumorigenesis in vivo

**Fig. 8:**
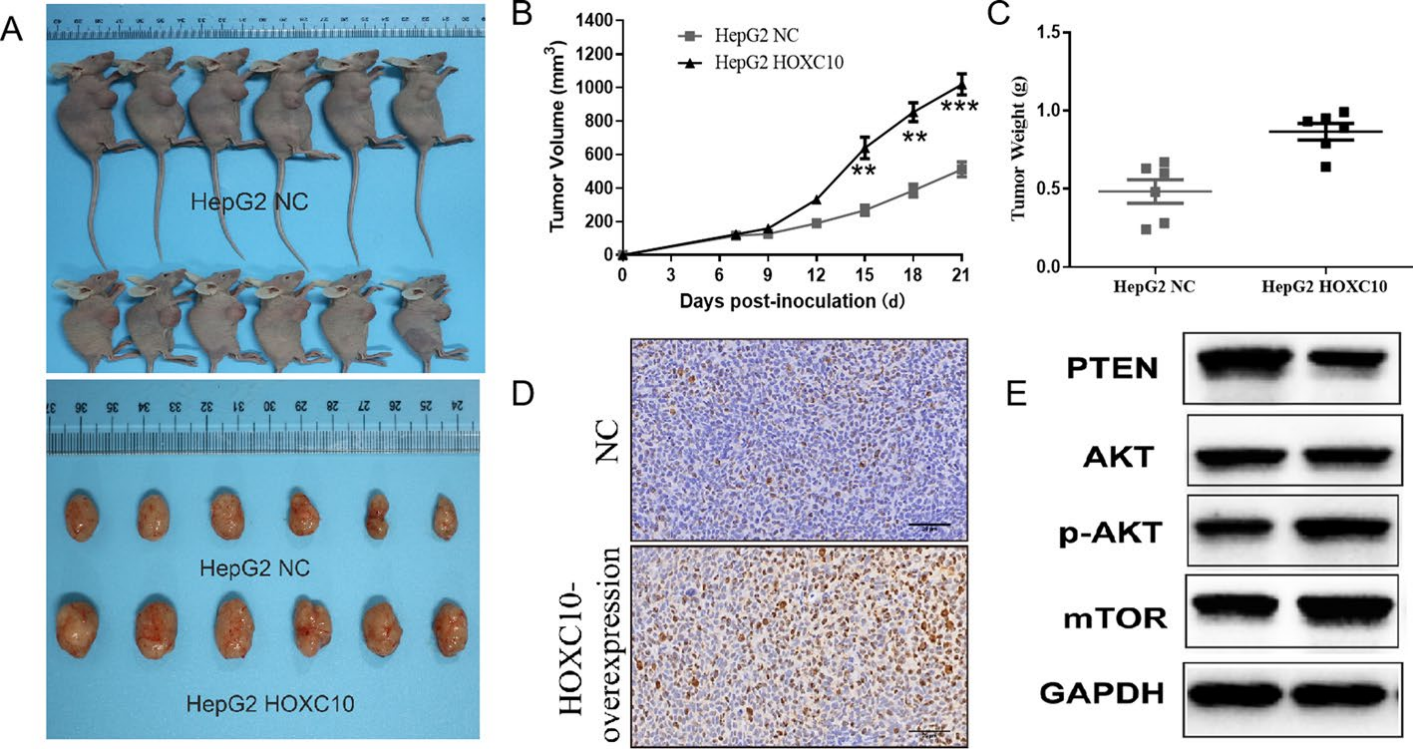
HOXC10 overexpression in HepG2 cells accelerated tumor growth in vivo. Tumor growth was observed 25 days after injection. **A** HOXC10 overexpression promoted tumor growth in the nude mice xenograft model in vivo. **B-C** Tumor volume and weight were also measured after HOXC10 overexpression. **D** The expression of Ki67 in tumor samples of HCC xenograft mice was detected by IHC. **E** The expression of PTEN, AKT, p-AKT, and mTOR in xenografts from the nude mice was determined using western blotting analysis.**P < 0.01, ***P < 0.001 (compared with HepG2 NC cells)

To further verify the function of HOXC10 in tumorigenesis in vivo, xenograft tumor model assays were performed by subcutaneously injecting the HepG2 variant with or without stable HOXC10 overexpression into the dorsal flank of nude mice. As shown in Fig. 8A, HOXC10 overexpression accelerated tumor growth in mice compared with the control group in mice. Furthermore, the tumor volume and weight after treatment were significantly larger than in the control group (Fig. 8B, C). Additionally, it was observed from IHC results that the expression level of Ki67 in HCC tumor tissues of the xenografted mice was markedly increased in HOXC10 overexpression mice compared with the control group (Fig. 8D). Western blotting results from tumor xenograft samples showed PTEN expression level was decreased and p-AKT and mTOR expression was significantly increased in tumors induced by HOXC10 overexpression (Fig. 8E). Consistent with the in vitro results, these data indicated that high HOXC10 expression was associated with the activation of the PTEN/AKT/mTOR signaling pathway in HCC in vivo.

## Discussion

In the present study, we first performed IHC detection of HOXC10 in 76 HCC tissues and then evaluated the relationship between HOXC10 expression and the clinicopathological features in HCC. Results showed that high expression of HOXC10 in the tumor tissue was found associated with poor prognosis. Patients with high HOXC10 expression showed a significantly lower OS rate. Consistently, similar results were demonstrated from the HOXC10 expression examination in HCC cell lines. HOXC10 was overexpressed in Huh7 cells and less expressed in the well differentiated cell line HepG2. HOXC10 overexpression significantly increased cell growth in vitro and in vivo, decreased apoptosis, and increased invasion and migration of HCC cells in vitro. However, HOXC10 knockdown in Huh7 cells showed the opposite changes. M^6^A plays an important role in posttranscriptional gene regulation [17]. Thus, we further explored the effect of m^6^A modification on HOXC10 expression.We showed that METTL3-mediated m^6^A modification enhanced HOXC10 expression by enhancing its mRNA stability. Furthermore, the in vitro and in vivo results showed that overexpressed HOXC10 activated the PTEN/AKT/mTOR pathway.

The gene plays an important role in cellular identity and embryonic morphogenesis during development [18, 19]. Furthermore, HOXC10 was reportedly highly expressed in many human cancers, such as gastric cancer, lung cancer, multiple myelomas, oral squamous cell carcinoma, and HCC [20–24]. In addition, HOXC10 has been shown involved in tumor proliferation, migration, and invasion in numerous studies [25–27]. In the present study, HOXC10 promoted proliferation and invasion in HCC, which is consistent with Dang’s report that upregulated HOXC10 induced by IL-1β promotes HCC metastasis [28]. Conversely, in a study by Ma, HOXC10 was suggested to serve as a critical negative regulator of cell proliferation via activation of the MAPK signaling pathway [29]. This discrepancy regarding the function of HOXC10 in liver cancer may be associated with the heterogeneity of tumors and different downstream signaling pathways.

To determine the potential mechanisms underlying HOXC10 promoting tumorigenesis in HCC cells, the effect of HOXC10 expression on the PTEN/AKT/mTOR signaling pathway was investigated. The role of the PTEN/AKT/mTOR pathway in HCC processes has attracted significant attention. For example, Wang found that activated cdc42-associated kinase 1 may promote HCC development via the PTEN/AKT/mTOR pathway [30]. Similarly, Su reported the PTEN/AKT/mTOR pathway might play a key role in the recurrence and prognosis of HCC [31]. However, whether this signal pathway is associated with HOXC10 remains unclear. In the present study, upregulation of HOXC10 decreased the PTEN expression and activated proteins in the AKT/mTOR pathway, including p-AKT and mTOR in vivo and in vitro. The opposite results were obtained with knockdown of HOXC10 using siRNAs in Huh7 cells. Overall, these findings reveal a novel molecular mechanism of PTEN/AKT/mTOR signaling pathway activation by HOXC10 in HCC.

## Conclusion

**Fig. 9:**
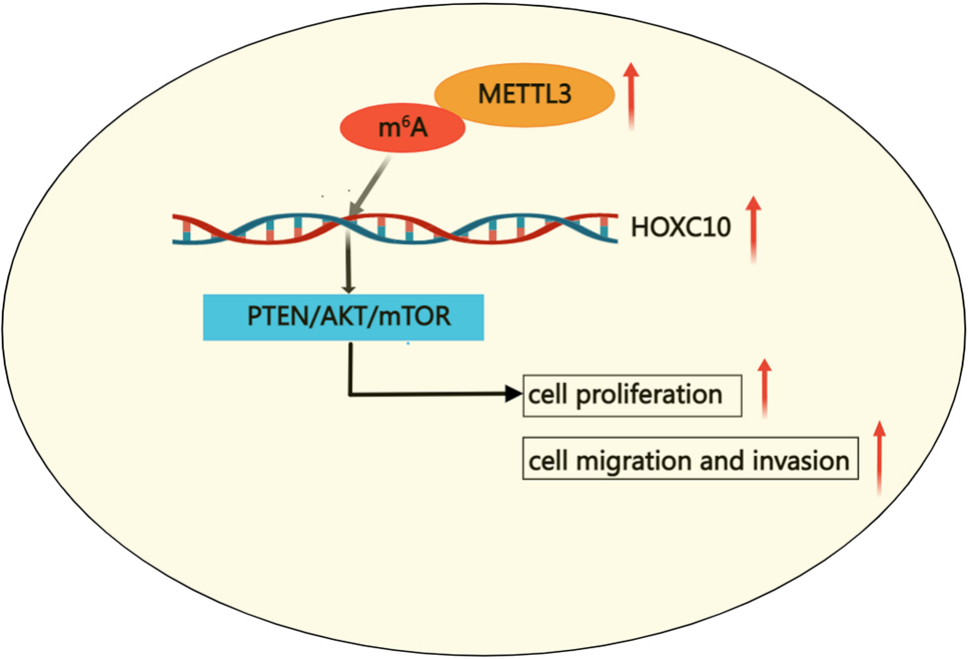
The graphical summary of the function and mechanism of HOXC10 in HCC

In conclusion, our study demonstrates that HOXC10 is a potential prognostic biomarker for HCC patients. We also provide evidence that METTL3-mediated upregulation of HOXC10 in HCC could promote cell proliferation and migration and invasion. Furthermore, HOXC10 function in HCC cells might be associated with the modulation of PTEN/AKT/mTOR signaling pathway (Fig. 9). Therefore, HOXC10 may be a promising therapeutic target for HCC treatment.

### Supplementary Information

Additional file 1: (DOC 18 KB)

## Data Availability

Materials described in the manuscript, including all relevant raw data, will be freely available to any researcher wishing to use them for non-commercial purposes, without breaching participant confidentiality.

## Abbreviations

HOX: Homeobox
HCC: Hepatocellular carcinoma
M^6^A: N6-methyladenosine
PVDF: Polyvinylidene difluoride
siRNA: Small interfering RNA
PI: Propidium iodide
PFA: Paraformaldehyde
AKT/mTOR: Protein kinase B/mammalian target of rapamycin
