# Supplementary material for: M6A-mediated upregulation of HOXC10 promotes human hepatocellular carcinoma development through PTEN/AKT/mTOR signaling pathway
Source: Discov Oncol. 2023 Sep 21;14:175. doi: 10.1007/s12672-023-00786-0 (PMC10514025; doi:10.1007/s12672-023-00786-0)
Supplement: Supplementary file 1 — Additional file 1: (DOC 18 KB) [file 12672_2023_786_MOESM1_ESM.doc]

| Name Sequence |
| --- |
| HOXC10 ACGAAGCGAAAGAGGAGATAAAG Sense (5’->3’)  CCAGCGTCTGGTGTTTAGTATAG Antisense (5’->3’)  GAPDH TCAAGAAGGTGGTGAAGCAGG Sense (5’->3’)  TCAAAGGTGGAGGAGTGGGT Antisense (5’->3’)  HOXC10- siRNA995 GGAGAUUAGCAAGACCAUUTT Sense (5’->3’)  AAUGGUCUUGCUAAUCUCCTT Antisense (5’->3’)  METTL3-siRNA GCUACCGUAUGGGACAUUATT Sense (5’->3’) UAAUGUCCCAUACGGUAGCTT Antisense (5’->3’)  HOXC10 Position-2,3 AACACCTATCCGTCCTACCT Sense (5’->3’)  CAGACATTCTCCTCCTTGACAC Antisense (5’->3’) |

Table S1. Primer sequences
